# On the formalization and reuse of scientific research

**DOI:** 10.1098/rsif.2011.0029

**Published:** 2011-04-13

**Authors:** Ross D. King, Maria Liakata, Chuan Lu, Stephen G. Oliver, Larisa N. Soldatova

**Affiliations:** 1 Department of Computer Science, Aberystwyth University, Wales SY23 3DB, UK; 2 Department of Biochemistry, Cambridge Systems Biology Centre, University of Cambridge, Sanger Building, 80 Tennis Court Road, Cambridge CB2 1GA, UK

**Keywords:** semantic web, logic, *Saccharomyces cerevisiae*, ontology

## Abstract

The reuse of scientific knowledge obtained from one investigation in another investigation is basic to the advance of science. Scientific investigations should therefore be recorded in ways that promote the reuse of the knowledge they generate. The use of logical formalisms to describe scientific knowledge has potential advantages in facilitating such reuse. Here, we propose a formal framework for using logical formalisms to promote reuse. We demonstrate the utility of this framework by using it in a worked example from biology: demonstrating cycles of investigation formalization [*F*] and reuse [*R*] to generate new knowledge. We first used logic to formally describe a Robot scientist investigation into yeast (*Saccharomyces cerevisiae*) functional genomics [*f*_1_]. With Robot scientists, unlike human scientists, the production of comprehensive metadata about their investigations is a natural by-product of the way they work. We then demonstrated how this formalism enabled the reuse of the research in investigating yeast phenotypes [*r*_1_ = *R*(*f*_1_)]. This investigation found that the removal of non-essential enzymes generally resulted in enhanced growth. The phenotype investigation was then formally described using the same logical formalism as the functional genomics investigation [*f*_2_ = *F*(*r*_1_)]. We then demonstrated how this formalism enabled the reuse of the phenotype investigation to investigate yeast systems-biology modelling [*r*_2_ = *R*(*f*_2_)]. This investigation found that yeast flux-balance analysis models fail to predict the observed changes in growth. Finally, the systems biology investigation was formalized for reuse in future investigations [*f*_3_ = *F*(*r*_2_)]. These cycles of reuse are a model for the general reuse of scientific knowledge.

## 1. Introduction

### 1.1. The state-of-the-art in recording biological research

Scientific research should be recorded with sufficient detail and semantic clarity to enable the information obtained from one investigation to be re-used in future investigations. The traditional way of recording science, based on the use of natural language, does not fully promote reuse as it permits too much ambiguity [1–3].

There are now a growing number of domain-specific data reporting standards for experimental data, especially in biology. These ensure that common experimental metadata are recorded, and partially deal with the ambiguity of natural languages by using standard taxonomies. The Minimum Information for Biological and Biomedical Investigations (MIBBI) project provides a resource for the existing checklists and fosters coordinated development [4]. These checklists are intended to promote transparency in experimental reporting, enhance accessibility to data and support effective quality assessment, thereby increasing the value of a body of work. Often the terminology used in checklists is supplied by a relevant ontology that formally defines those terms. The Minimum Information about a Microarray Experiment (MIAME) was the original checklist [5], and the MGED Ontology provided definitions for the MIAME terms. There now exists many other similar standards [6].

An important limitation of these standards is that they are focused on the annotation of experimental data for a specific domain. This results in both duplication of effort, and different standard representations for the same piece of knowledge. Another important limitation is that they are focused on the annotation of experimental data, and they therefore do not pay enough attention to the rest of the scientific process. These limitations have led to the development of more general ontologies to provide a framework for recording not only experimental data with a limited set of associated metadata, but all essential information about biological experiments. The EXPO (a generic ontology of scientific experiments) ontology formalizes domain-independent knowledge about the organization, execution and analysis of scientific experiments [7]. The more recent OBI (the Ontology for Biomedical Investigations) project (http://obi-ontology.org) aims to model the design of an investigation: the protocols, the instrumentation, and materials used in experiments and the data generated [8]. Ontologies such as EXPO and OBI enable the recording of the whole structure of scientific investigations: how and why an investigation was executed, what conclusions were made, the basis for these conclusions, etc. As a result of these generic ontology development efforts, the Minimum Information about a Genotyping Experiment (MIGen) recommends the use of terms defined in the Ontology for Biomedical Investigations (OBI). If other checklists follow the same approach—the use of a generic or a compliant ontology to supply terms—then this will stimulate cross-disciplinary data-sharing and reuse [9].

The desire to record as much detail about an investigation as possible in order to make the investigation more reproducible, and reusable, needs to be balanced against the practicality of persuading scientists to actually record the details. It is these ‘human factors’ that are in large part the reason for the restricted nature of most existing reporting standards: they are a compromise between what is reasonable to expect a working scientist to record, and what one would ideally like to record. It is hoped that, over time, the added value to science of comprehensive data will alter the behaviour of working scientists such that they will be prepared to put greater effort into the formal reporting of scientific investigations, and that better tools will be built which more easily facilitate this recording process.

### 1.2. Robot scientists

The investigations described in this paper arose out of research into the automation of scientific research. For over 10 years we have been developing ‘Robot scientists’: these are physically implemented computer/robotic systems that use techniques from artificial intelligence (AI) to execute cycles of scientific experimentation [10]. A Robot scientist is designed to automatically originate hypotheses to explain observations, devise experiments to test these hypotheses, physically run the experiments using laboratory robotics, interpret the results and then repeat the cycle.

The development of Robot scientists is significant for the reuse of scientific investigations because with Robot scientists, unlike human ones, the production of comprehensive metadata about their investigations is a natural by-product of the way they work. Everything they do can be made explicit, and this enables all aspects of a scientific investigation to be recorded, and potentially re-used. This advantage of Robot scientists over human ones makes the records of the science they produce of higher quality and easier to re-use. It also makes Robot scientists excellent test beds for the development of new approaches to the recording and reuse of scientific investigations.

We have recently developed the Robot scientist ‘Adam’ to automate yeast (*Saccharomyces cerevisiae*) functional genomics investigations [11]. Adam's hardware is designed to execute high-throughput micro-batch growth experiments using microtitre plates. Adam measures growth curves (phenotypes) of selected microbial strains (genotypes) growing in defined media (environments). Adam's investigations are recorded in great detail making them suitable for the testing of new approaches to the recording and reuse of scientific investigations.

To demonstrate the full automation of a Robot scientist, we programmed Adam to repeat the experiments of our first, semi-automated, Robot scientist [10]. These experiments concern the rediscovery of functional genomics knowledge about the aromatic amino acid biosynthesis pathway in *S. cerevisiae*. The comparison between the previous gene-function prediction experiments and those performed by Adam showed that results for Adam were slightly better than the original Robot scientist. This demonstrates that cycles of experiment can be automated by a Robot scientist, and confirms the first Robot scientist's results.

We also applied Adam to the discovery of genes encoding orphan enzymes in *S. cerevisiae*—enzymes catalysing biochemical reactions believed to occur in the yeast cell, but for which the gene encoding the relevant enzyme has not been identified. Note that the discovery of the genes encoding these enzymes is presumably particularly difficult, as decades of research had not found them. Adam formulated and tested 20 hypotheses concerning genes encoding 13 orphan enzymes. The weight of the experimental evidence for the hypotheses varied, and 12 novel hypotheses were confirmed with *p* < 0.05 for the null hypothesis. We argue that Adam's confirmation of these 12 hypotheses it formed constitutes the first example of novel scientific knowledge generated by a machine [11].

### 1.3. The laboratory ontology for Robot scientists

To formalize Adam's functional genomics experiments, we developed the LABORS ontology (LABoratory Ontology for Robot Scientists) [11]. LABORS is a version of the ontology EXPO [7] customized for Robot scientists. (For clarity, below, we use italics for terms in the ontology where appropriate.) In order to support a comprehensive representation of scientific investigations, LABORS defines various structural research units, e.g. trial, study, cycle of study and replicate (see the definitions and explanations in Qi *et al*. [12]). All aspects of the scientific process (i.e. hypotheses formation, experiment planning and analyses of results) have to be consistently represented in a form that can be processed by a robot. LABORS also defines what is the most essential information about automated investigations, i.e. design strategy, plate layout, expected results and actual results. (Robot scientists could potentially record absolutely all information about implemented investigations, i.e. all movements of the robots. However, it is important to record only essential information and in a structured way for further processing.) Finally, LABORS defines the concepts and relations in the functional genomics data (e.g. optical density readings and growth curves) and metadata (i.e. temperature, humidity, time-stamp and investigator).

The application of an ontology to describe a particular scientific investigation results in a logical description of that investigation. Philosophers generally agree that scientific knowledge is best expressed using formal logical languages [13]. The advantages of logic are of increasing practical importance as logic, and especially description logic, are being used more frequently and more to describe biological knowledge (e.g. [14]).

LABORS is expressed in the W3C standard Web Ontology Language OWL-DL [15]—a form of description logic. LABORS uses EXPO as an upper level ontology and OBO RO as a set of relations. The instances of the classes are stored in a relational database. LABORS is expressed in the W3C Semantic Web Ontology Language OWL-DL. It has been checked for logical consistency with the reasoner FaCT++. Unfortunately, reasoners for description logic are still inefficient. We therefore translated both LABORS and the corresponding database into Datalog in order to use the SWI-Prolog reasoner for required applications. Datalog enables search, querying, retrieval and automated reasoning. We are continuing to investigate the use of semantic web technology [16], i.e. triplestore as an alternative to Datalog databases.

## 2. Results

A major motivation for developing the formalization used for Adam's functional genomics investigations is the expectation that its use should make an investigation more easily re-usable. An ontology-based formalization makes it possible to keep an accurate track of all the result units used for different goals, while preserving the semantics of all the experimental entities involved in all the investigations. Therefore, it is possible to safely reuse information without fear that the meaning of the information will subtly depend on the context in an undocumented way. In addition, thanks to the comprehensive nature of the formalism, it is possible to safely re-use the information without fear that important information is missing. For example, it is possible to check if two yeast strains were grown under the same experimental conditions (temperature, medium, etc.), and if the same methods were used to calculate growth parameters, etc. Formalization makes it easier to compare like with like, and decreases the chance of the introduction of systematic error into a new investigation based on reusing information from another.

Below we propose a formal framework for using logical formalisms to promote reuse. We then demonstrate the utility of this framework by employing it in a worked example from biology: demonstrating cycles of investigation formalization and reuse to generate new knowledge.

### 2.1. A generic framework for the reuse of investigations

We will refer to the process of formalization as *F*, and we will denote particular instances of the formalism *F* applied to particular investigations *x* and *y* as *f*_*x*_ = *F*(*x*), *f*_*y*_ = *F*(*y*), etc. We will denote the process of reusing a particular investigation as *R*. Particular instances of reuse *R* applied to a particular formalized investigation represented as *f*_*i*_ within another investigation with different goals will be denoted as *r*_*i*_ = *R*(*f*_*i*_).

We propose the following formal generic framework for formalizing the reuse of knowledge in scientific investigations:

#### 2.1.1. Formalization (F)

— There is a formalism *F* for recording the most essential components of a scientific investigation.
— There are domain-specific formalisms *D*_1_, *D*_2_, *D*_3_, … that are compliant with *F* and that formalize domain-specific entities.
— An investigation is recorded through the use of the formalism *F*, and the corresponding domain-specific formalisms *D*_*i*_, *D*_*j*_, … . The formalisms *F*, *D*_*i*_, *D*_*j*_, … form a system of formalisms Ext(*F*).
— There is a set of completed investigations formalized using the *F*-compliant formalisms Ext(*F*): *f*_1_, *f*_2_, *f*_3_, … .
— There is a set of queries defined over the terms in the formalisms Ext(*F*)*, Q*(Ext(*F*)): *q*_1_, *q*_2_, *q*_3_, … . These queries *Q* can be applied to the formalized investigations *f*_1_, *f*_2_, *f*_3_, … .

#### 2.1.2. Reuse (R)

— There is a new investigation *i* which has as one of its objectives to re-use data and knowledge items from the completed investigations formalized as *f*_1_, *f*_2_, *f*_3_, … .
— The objects, goals and hypotheses of the investigation *i* are specified in the terms defined within the formalism Ext(*F*).
— The entities used to compose the objectives, goals and hypotheses of the investigation *i* are compared with the entities used to compose the hypotheses and results of the completed investigations, which are recorded as Ext(*F*): *f*_1_, *f*_2_, *f*_3_, … . The matches found *M*, if any, are output as *M*: *m*_1_, *m*_2_, *m*_*3*_*,* … .
— If there are not any matches *M* found, then reuse of the previous investigation *r*_*i*_ = *R*(*f*_1_, *f*_2_, *f*_3_, …) cannot be supported by the formalism Ext(*F*).
— Queries *Q*(*M*): *q*_1_, *q*_2_, *q*_3_, … are run over the set of completed investigations which are formalized as Ext(*F*): *f*_1_, *f*_2_, *f*_3_, …. The outputs of the queries *Q*(*M*) provide knowledge items and data which can be re-used *r*_*i*_ = *R*(*f*_1_, *f*_2_, *f*_3_, …) in order to achieve the goals and objectives of the investigation *i*.

#### 2.1.3. Formalization of reuse *F*(R)

— The investigation *i* which includes the reuse *r*_*i*_ = *R*(*f*_1_, *f*_2_, *f*_3_, …) can be formalized with the formalism Ext(*F*): *f*_*i+*1_ = *F*(*r*).

### 2.2. The formalization of cycles of reuse

It is possible to have cycles of formalizations and reuses. For example, *r*_1_ = *R*(*f*_1_) represents the reuse of formalism *f*_1_. The investigation based on reuse of formalism *f*_1_ could then be formalized *f*_2_ = *F*(*r*_1_). This formalization could then be re-used *r*_2_ = *R*(*f*_2_), *ad infinitum*. This models cycles of formalization and reuse as a mutually recursive process.

Below we describe an example of cycles of formalizations and reuse, where LABORS serves as the formalism *F* for recording the key components of scientific investigations. The set of queries *Q* is defined by the list of LABORS terms (plus instances of the classes defined in LABORS and stored in a relational database), relations between those terms and the syntax of the SWI-Prolog inference engine that was used for querying. The full logical representations of all the formalization may be found at http://www.aber.ac.uk/en/cs/research/cb/projects/robotscientist/results/.

This example of cycles of formalizations and reuse demonstrates that LABORS is a suitable formalism to support the reuse of scientific research results. The described reuse investigations have generated new scientific results, not through executing new experiments, but by reusing the results of the previous experiments.

#### 2.2.1. Formalization *f*_1_

**Figure 1.**
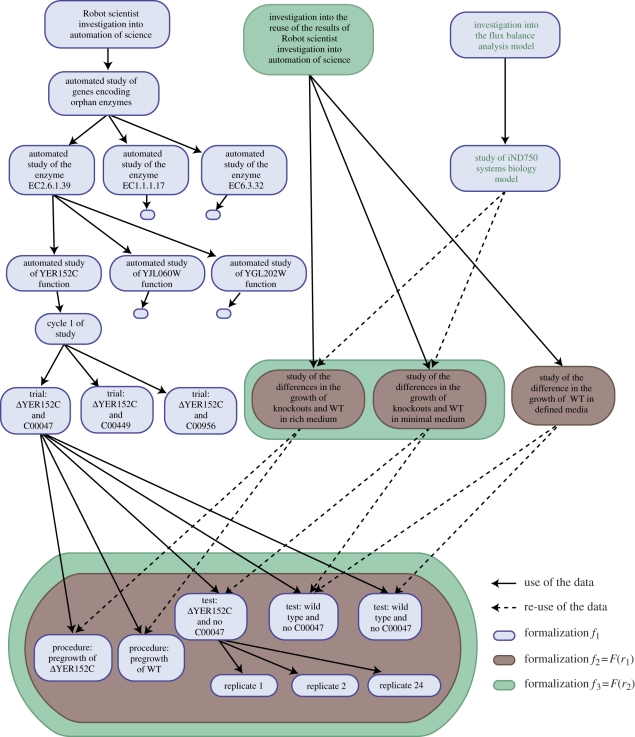
Overall structure of the formalization (a fragment). The figure shows three investigations: the investigation into automation in science (in blue), the investigation into the reuse of the results of the investigation into automation in science (in brown) and the investigation into the FBA model (in green). The boxes represent parts of the investigations, the links are has-part relations.

We first used LABORS to formalize Adam's functional genomics investigation (investigation-1 = ‘Robot scientist investigation into automation of science’; figure 1) [11,17]. The National Center for Biomedical Ontology (NCBO) taxonomy was used as a formalism *D* to define the object of the functional genomics investigation—*S. cereviceae*. This class was imported from NCBO to LABORS, thus Ext(*F*) *= F* = LABORS in this case. This formalization resulted in the logical description *f*_1_, involving 9312 research units (segments of experimental research such as investigations, studies, tests, trials, replicates).

This description has a nested tree-like structure, 10 levels deep, that logically connects the experimental observations to the experimental metadata. A typical path through the formalization would proceed as follows: the *investigation* into the automation of science has a part (the *investigation* into whether the Robot scientist Adam can discover some novel science that has a part (the *study* aimed at finding the genes encoding orphan enzymes, which has a part (the *study* of the orphan enzyme E.C.2.6.1.39 in *S. cerevisiae*, which has a part (the *cycle of study* of the gene *YER152C*, which has a part (the *study* of the gene *YER152C*, which has a part (the *cycle* 1, which has a part (the *trial* of the compound C00047/lysine, which has a part (the *test* of addition of C00047/lysine, which has a part (the *replicate* 1, which has more than 300 observations))))))))). This complete structure resembles a computer program trace and takes up several hundred megabytes. We believe that this formalization of the functional genomics investigation is the most detailed and complete description of any substantial scientific investigation.

#### 2.2.2. Reuse *r*_1_ = *R*(*f*_1_)

LABORS is designed to support the reuse of investigations. Within LABORS, the class investigation is defined as a subclass of the class process and it can have parts such as studies, trials, tests, replicates which are also subclasses of the class *process*. This *representation* of investigations facilitates the construction of new *investigation*s by reusing parts of existing investigations. LABORS uses the *part_of* relation from the OBO Relation Ontology. It is the inverse to the relation *has_part* and is a transitive relation: if a test is a part of a study, and the study is a part of an investigation, then the test is a part of the investigation. Research units from completed investigations can thereby be defined via *part_of* relations as parts of new investigations. Moreover, research units can include input and output information. In this way, results from a completed research unit, e.g. hypotheses, can be used as input for other research units. Many queries, which researchers already routinely apply, can be considered as very simple reuse queries, for example ‘What is known about a particular compound?’. Such queries are supported by LABORS, and we argue that other ontological formalisms are less supportive of data reuse.

To investigate the utility of formalization for re-using information from scientific investigations, we re-used the formalization *f*_1_ of the functional genomics investigation to investigate yeast phenotypes (investigation-2 = ‘investigation into the reuse of the results of Robot scientist investigation into automation of science’; figure 1). In this reuse investigation, we investigated the relationship between *S. cerevisiae* genotype, environment and phenotype [18]. This investigation has as an objective to re-use data and information from the investigation-1 in order to understand the impact of gene deletions on yeast growth in rich and minimal media. Investigation-2 had two parts. The first was a study of the differences in growth of deletant (gene removed) and wild-type (no gene removed) strains in the same media: we varied the genotype while keeping the environment constant. The hypotheses of the investigation-2 are expressed using the terms defined in LABORS (as textual entities):

there is a difference in growth between knockouts

and wild-type in rich medium;

there is a difference in growth between knockouts

and wild-type in minimal medium;

there is a difference in growth of wild-type in

different media.

Hypotheses may be instantiated, e.g. there is a difference in growth between *Δ*YER152C and wild-type in rich medium (for strain name formalisms see [11]) and also expressed as logical entities:

difference_growth(delta_YER152C, wt).

has_object(Research_unit, s_ cerevisiae).

has_participant(Research_unit, rich_medium).

has_participant(Research_unit, delta_YER152C).

has_participant(Research_unit, wt).

The SWI-Prolog engine can run queries specified by the hypothesis expressed as a logical entity to find all research units (studies, trials, tests, replicates) that have *S. cerevisiae* as an object of study, involve the yeast strain *Δ*YER152C, involve the yeast strain wild-type and use rich medium as an environment. In the same way, it is possible to select research units that have identical experimental designs, normalization strategies, etc. The list of yeast strains served as matches *M*: *m*_1_, *m*_2_, *m*_3_, … Example queries are:

has_participant(Research_unit, wt).

is_concretised_as(Research_unit,

experiment_design).

Such queries identify which data, from which research units, may be re-used in order to achieve the objectives of investigation-2. Such optical density (OD) data were from pre-growth experiments and from trials and their replicates (figure 1).

**Figure 2.**
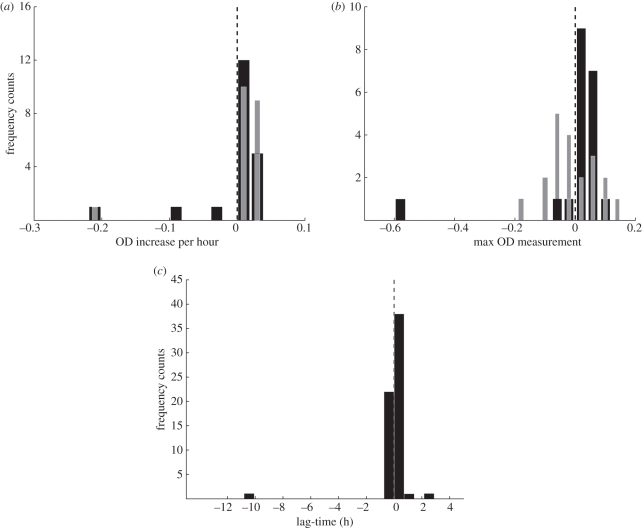
(*a*) The histogram shows the median observed differences in the growth rate of a knockout strain (k) and that of the wild-type (w) in both minimal and rich media; we used medians as they are more robust to outliers (black bars, k-w in minimal; grey bars, k-w in rich). (*b*) The histogram shows the median observed differences in global maximum OD between the knockout strain (k) and that of the wild-type (w) in both minimal and rich media (black bars, k-w in minimal; grey bars, k-w in rich). (*c*) The histogram shows the median observed differences in hours between the lag-time parameter of the wild-type grown in the presence of a nutrient and that of the wild-type grown on minimal medium (black bars).

We compared 20 different single-gene deletion genotypes with the wild-type using both a rich and a minimal growth medium. The deleted genes are from a little-studied class: non-essential (not required for growth on rich medium) and isoenzymes (table 1). The second part was a study of the differences in growth of the wild-type in different media: we varied the environment while keeping the genotype constant. We compared the growth of the wild-type on 63 different growth media [11]. Figure 2 summarizes the results of the reuse study into yeast phenotype.

**Table 1.** Comparison of the predicted (sim.) change in growth rate (deletant − wild-type) with the experimentally measured (exp) growth rate change for the 20 manually studied gene deletants. MM denotes minimal medium; YPD is rich medium; n.a. means the reactions are not present in the iND750 model.

reaction ID in iND750	deleted gene (open reading frame)	exp. DM	sim. DM	exp. YPD	sim. YPD
R_AATA	YER152C	0.009	−0.733	0.019	−0.222
R_AATA	YGL202W	−0.024	−0.733	0.024	−0.222
R_AATA	YJL060W	0.013	−0.733	0.024	−0.222
R_AGAT_SC	YDL052C	0.009	−0.733	0.034	−0.805
R_FTHFCLm	YER183C	0.022	0	0.014	0
R_G6PDA	YGR248W	0.017	0	0.007	0
R_G6PDA	YHR163W	−0.222	0	0.005	0
R_G6PDA	YNR034W	0.023	0	0.028	0
R_GLUN	YIL033C	−0.079	0	−0.205	0
R_M1PD	YNR073C	0.016	0	0.024	0
R_MACACI	YLL060C	0.011	0	0.014	0
R_POLYAO2	YMR020W	0.016	0	0.023	0
R_PUNP1	YLR017W	0.003	0	0.008	0
R_PUNP1	YLR209C	0.017	0	0.004	0
R_PYDXK	YNR027W	0.013	0	0.023	0
R_PYDXK	YPR121W	0.036	0	0.025	0
R_SERATi	YJL218W	0.015	−0.733	0.038	0
n.a.	YDL168W	0.018	0	0.024	0
n.a.	YJL045W	0.016	0	0	0
n.a.	YLR070C	0.012	0	0.019	0

Figure 2*a* shows the difference in maximum growth rate (*μ*_max_) between the deletant strains and the wild-type. In both the rich and the defined media, the general shape of the distributions is similar—a distinct mode and a skew to the left. The two distributions modes (and medians) are greater than zero, which means that (on average) the deletant strains grew faster than the wild-type (they are tachytrophs); the skew to the left indicates that there were some deletant strains that grew much slower than the wild-type (bradytrophs). In moving from the rich to the defined medium, the distribution shifts to the left, and the left tail extends. This means that the deletant strains grew (on average) less well, relative to the wild-type, than on the defined medium and a subset of them grew much worse.

Figure 2*b* shows the difference between maximum cell density (biomass yield) between the deletant strains and the wild-type. On the rich and the defined media, the observed distributions are qualitatively different. In the rich medium, both the mode and the median are less than zero, which means that (on average) the deletants' maximum cell density was lower (they are ischnotrophs) than that of the wild-type, and there is little skew. In the defined medium, both the mode (and median) shift to the right and are greater than zero, which means that the deletant strains produced relatively more biomass (on average) than the wild-type, on the defined medium (they are pachytrophs). The outlier is the deletant strain *Δ*SOL3—this was the strain most affected, relative to the wild-type, by the addition of metabolites [11].

Figure 2*c* shows the difference in lag-time of the wild-type moving from rich medium into the standard defined medium when compared with the defined medium with added metabolites. Here the mode is greater than zero, which means that (in general) the addition of a metabolite increases the lag-time. This was unexpected, and may reflect changes in the physico-chemical environment (e.g. pH) owing to the addition of the metabolite, and the consequent need for the cells to adapt to the new environment.

It is surprising that the removal of non-essential enzymes generally results in enhanced growth (both higher maximum growth rates (figure 2*a*) and higher maximum cell densities (figure 2*b*) as it is often assumed that growth of the wild-type is optimal—but see [19].

#### 2.2.3. Formalization *f*_2_ = *F*(*r*_1_)

We used LABORS to formalize investigation-2 = ‘investigation into the reuse of the results of Robot scientist investigation into automation of science’. A fragment of the formalism *f*_2_ is shown in figure 1. It shows, for example, that information from the two *test*s: ‘*Δ*YER152C and no C00047’ and ‘wild-type and no C00047’ from the investigation represented as *f*_1_ were re-used in the ‘study of the difference in the growth of knockouts and WT in minimal medium’. (Each of these tests has 12 replicates and hundreds of observations logically associated to it in the database.) Reuse therefore enabled observational data collected to answer questions about yeast functional genomics to also answer questions concerning the relationship between genotype, environment and phenotype. These questions are quite different from those concerned with functional genomics, for which the original investigations were designed to answer.

The investigation into yeast phenotypes also re-used data/metadata from the functional genomics investigation that was recorded, but not directly used to infer the functional genomics investigation's conclusions. These data/metadata describe how well the deletant strains grew on the rich medium, YPD. For example, the two *procedure*s: ‘pregrowth of *Δ*YER152C’ and ‘pregrowth of WT’ were re-used in the ‘study of the difference in the growth of knockouts and WT in rich medium’ along with their *replicate*s and observations (figure 1). Each of these *procedure*s has 48 replicates plus the hundreds of observations logically associated with them. This pregrowth information was irrelevant to the functional genomics study as it was part of the preparation for inoculation, and the inoculum size for the main investigation was subsequently normalized. Use of this information to answer new questions illustrates the importance of formally describing and making available all research undertaken, and not just research that is used directly in a scientific paper's conclusions, as it may prove useful in other investigations.

#### 2.2.4. Reuse *r*_2_ = *R*(*f*_2_)

To demonstrate cycles of formalization and reuse, we re-used information from investigation-2 = ‘investigation into yeast phenotype’ formalized as *f*_2_ in an investigation into systems biology modelling (investigation-3 = ‘investigation into the flux balance analysis (FBA) model’; figure 1). FBA modelling [20] is currently the most common quantitative approach in systems biology to modelling metabolism. It is a constraint-based approach that uses linear programming to identify a flux distribution that optimizes a given objective function. The output of an FBA model for a specified growth medium is an estimated maximum growth rate. The yeast FBA model we used was based on the iND750 model of Duarte *et al*. [21]. We re-used the formalization of yeast phenotype investigation to investigate the suitability and accuracy of FBA models of *S. cerevisiae* metabolism. Investigation-3 has the following goals (expressed as text entities):

to test the suitability of FBA models to predict

yeast phenotypes.

to test the accuracy of FBA models.

In order to achieve these goals, data and information from the already completed and formalized investigation-2 were re-used. The hypotheses of investigation-3 are encoded as logical entities, e.g.

not_match(model_iND750_prediction,

data_reuse_results),

where the information item data_reuse_results serves as the match *M*, and was used to retrieve results of all research units within the the yeast phenotypes investigation of yeast strains. Figure 1 shows that information from the ‘study of the difference in the growth of knockouts and WT in rich medium’, and the ‘study of the difference in the growth of knockouts and WT in minimal medium’ were re-used in the ‘study of the iND750 Systems Biology model’.

For each of the 20 genes investigated within investigation-2, FBA modelling was used to simulate the change in the flux distribution associated with the deletion (table 1). Reactions associated with multiple genes could be annotated as either isoenzymes or enzyme complexes. For model prediction, both were considered—with only the results from enzyme complexes being reported here. To incorporate the hypothesized effect of the deletion of a gene, the associated reaction(s) were disallowed by setting the lower and upper bounds for their fluxes to zero. Four of these reactions are blocked and unable to carry any flux at all. For four other reactions, using the minimal medium, the flux range for minimal growth is large and encompasses zero. Therefore, the FBA model predicts that elimination of these eight reactions will not prevent the cell from growing using the defined medium. The model also predicts that the removal of two of the reactions (involving four gene annotations) will stop the cell growing, as the minimum growth requires their fluxes to be positive.

The estimated differences in growth rates between wild-type and deletant strains, re-used from the investigation into yeast phenotype, are generally inconsistent with the predictions of the FBA model (table 1). The reason is that many of the deletants were observed to grow at a higher maximal growth rate than the wild-type, and a central assumption of most FBA modelling is that metabolic fluxes are optimized to maximize cell growth. This means that if setting any flux to zero improved cell growth this would already have been found during optimization. This inconsistency could have been directly inferred without simulation. However, the simulation also illustrates that the quantitative differences predicted by the FBA modelling for the other deletants are also inconsistent with observations (table 1). We have confirmed these results using the minimization of metabolic adjustment approach to modelling deletion growth rate [22].

#### 2.2.5. Formalization 3 (*f*_3_ = *f*(*r*_2_))

We have demonstrated two cycles of formalization and reuse: *f*_1_, *r*_1_ = *r*(*f*_1_), *f*_2_ = *f*(*r*_1_), *r*_2_ = *r*(*f*_2_). In principle, these cycles can be repeated *ad infinitum*, reflecting the cumulative nature of scientific knowledge discovery. To continue the process, we therefore used LABORS again to formalize the systems biology investigation *f*_3_ = *F*(*r*_2_) (‘investigation into the FBA model’; figure 1). This formalization is available for further reuse of the information in future cycles of investigation: for example, an investigation that compares different systems biology models with FBA ones: *r*_3_ = *R*(*f*_3_), with the formalization of that investigation being *f*_4_ = *F*(*r*_3_), and so on.

## 3. Discussion

The proposed reuse framework is a principled way of reusing the existing knowledge from scientific investigations in new investigations. This framework obviously requires additional resources to implement compared with using no formalism. However, it is clearly generally cheaper, and faster, to re-use the existing knowledge from scientific investigations than to regenerate it afresh from wet biology investigations.

The vision we have of science is the comprehensive annotation of all investigations with metadata derived from standard ontologies and storage of these metadata and data in open repositories. This would make scientific knowledge more explicit, scientific results more reproducible, help detect errors, promote the interchange and reliability of experimental methods and conclusions, and remove redundancy. It would also enable accrued scientific knowledge to be re-used to answer other scientific questions. Scientific investigations that are comparable could be identified by their metadata, and then data-mining algorithms used to find patterns in them. These patterns could then be used to generate new hypotheses, which could be tested using other annotated investigations, or through new empirical research. In this paper, we have made a step towards this vision; we have shown that it is possible to demonstrate repeated cycles of formalization and reuse.

The EXPO and LABORS ontologies were developed when no other generic formalism for the logical description of experiments was available. The OBI project aims to provide such a formalism. OBI v.1 has been released recently (November 2010). The Robot scientist project joined the OBI project in October 2008, and the LABORS representations are aligned with the OBI representations. However, the reuse features discussed in this paper are still not inbuilt into OBI.

## 4. Conclusions

A comprehensively described scientific investigation is a permanent contribution to knowledge, and therefore improved ways of recording scientific investigations make the scientific process more efficient. The use of logical formalisms has clear theoretical advantages over using natural languages owing to their clear semantics and ability to represent all aspects of the scientific process. We have demonstrated their practicality for describing research through a worked example of cycles of formalization and reuse involving yeast biology. This has resulted in the improved understanding of the importance of non-essential enzymes when growing in standard defined media, and has also shown that FBA models fail both qualitatively and quantitatively to predict the observed changes in growth. The cycles of reuse in these investigations are a model for the general reuse of scientific knowledge.

Of course, the demonstration of the utility of the reuse formalization does not constitute proof that such logical formalisms are generally applicable and useful in practice for reuse, as that would require multiple test comparisons taken from multiple domains, which we hope will occur in due course. However, given their theoretical advantages, and the results presented here, we argue that the balance of evidence supports the case for using logical formalisms to describe research in order to promote its reuse.
